# The hydrolethalus syndrome protein HYLS-1 regulates formation of the ciliary gate

**DOI:** 10.1038/ncomms12437

**Published:** 2016-08-18

**Authors:** Qing Wei, Yingyi Zhang, Clementine Schouteden, Yuxia Zhang, Qing Zhang, Jinhong Dong, Veronika Wonesch, Kun Ling, Alexander Dammermann, Jinghua Hu

**Affiliations:** 1 Department of Biochemistry and Molecular Biology, Mayo Clinic, Rochester, Minnesota 55905, USA; 2 Key Laboratory of Insect Developmental and Evolutionary Biology, Institute of Plant Physiology and Ecology, Shanghai Institutes for Biological Sciences, Chinese Academy of Sciences, Shanghai 200032, China; 3 Max F. Perutz Laboratories, Vienna Biocenter (VBC), University of Vienna, A-1030 Vienna, Austria; 4 Division of Nephrology and Hypertension, Mayo Clinic, Rochester, Minnesota 55905, USA; 5 Mayo Translational PKD Center, Mayo Clinic, Rochester, Minnesota 55905, USA

## Abstract

Transition fibres (TFs), together with the transition zone (TZ), are basal ciliary structures thought to be crucial for cilium biogenesis and function by acting as a ciliary gate to regulate selective protein entry and exit. Here we demonstrate that the centriolar and basal body protein HYLS-1, the *C. elegans* orthologue of hydrolethalus syndrome protein 1, is required for TF formation, TZ organization and ciliary gating. Loss of HYLS-1 compromises the docking and entry of intraflagellar transport (IFT) particles, ciliary gating for both membrane and soluble proteins, and axoneme assembly. Additional depletion of the TF component DYF-19 in *hyls-1* mutants further exacerbates TZ anomalies and completely abrogates ciliogenesis. Our data support an important role for HYLS-1 and TFs in establishment of the ciliary gate and underline the importance of selective protein entry for cilia assembly.

## Abstract (web summary)

Transition fibres (TFs) and the transition zone (TZ) are basal ciliary structures thought to act as a gate to regulate protein transport. Here the authors show that the *C. elegans* orthologue of hydrolethalus protein 1, HYLS-1, disrupts both structures and compromises the trafficking of cargo through the ciliary gate.

## Graphical abstract

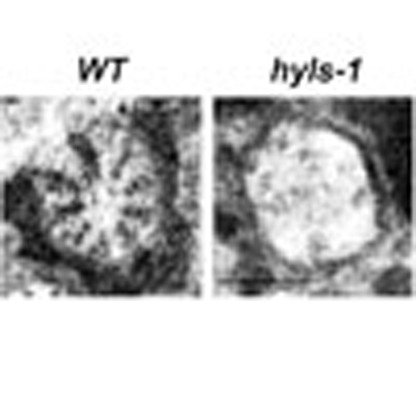

Primary cilia are sensory organelles emanating from the cell surface of most eukaryotic cells to perceive various environmental cues^1^,^2^. A wide spectrum of human disorders, collectively termed ciliopathies, has been linked to mutations in genes involved in cilia formation and/or function^3^,^4^,^5^. Unlike other membrane-enclosed cellular organelles, the ciliary lumen is open to the cytoplasm at the ciliary base. Mounting evidence suggests that gating mechanisms exist to regulate selective ciliary entry and exit of membrane and soluble proteins, whose coordinated function makes the cilium a distinct functional entity^6^,^7^,^8^. It has been proposed that transition fibres (TFs), which develop from the distal appendages of the mother centriole during ciliogenesis, and the transition zone (TZ) at the proximal-most segment of the axoneme, two structurally distinct and highly conserved subdomains, are functional compartments of the ciliary gate^9^,^10^.

The TZ is characterized by Y-links that connect the axoneme to the ciliary membrane. Dozens of ciliopathy proteins, including nephronophthisis (NPHP), Joubert syndrome and Meckel–Gruber syndrome (MKS) proteins, assemble into multimeric protein modules to regulate TZ integrity and function^10^. Loss of TZ integrity results in loss of the diffusional barrier to ciliary entry but does not significantly affect intraflagellar transport (IFT) or axoneme assembly in *Caenorhabditis elegans*^10^,^11^,^12^. Immediately proximal to the TZ, TFs anchor basal bodies to the apical membrane and constitute the first visible physical barrier between the cytoplasm and ciliary lumen^13^. In contrast to the TZ, only six proteins, CEP164, CEP83 (CCDC41), CEP89 (CCDC123), SCLT1, CEP128 and FBF1, have been identified as components of TFs or distal appendages^14^,^15^,^16^,^17^,^18^,^19^,^20^. Many of these proteins are poorly conserved in ciliated invertebrates that also possess TFs, suggesting additional components await identification^21^. Loss of TFs has been reported to abrogate basal-body-to-membrane docking in vertebrates^16^ and impair recruitment of ciliary membrane proteins^16^,^19^,^22^,^23^,^24^. Consistent with the essential role of TFs in the context of cilia, TF components and proteins regulating TF formation have been associated with cilia-related disorders. Thus, CEP164 and CEP83 have been found to be mutated in NPHP^25^,^26^, whereas SCLT1 and two centriole proteins critical for appendage formation, OFD1 and C2CD3, have been linked to oral–facial–digital syndrome^27^,^28^,^29^, and TTBK2, an interactor of CEP164, have been linked to spinocerebellar ataxia^30^.

Here we set out to examine the molecular mechanisms underlying TF assembly and function using *C. elegans* as an experimental model. We identify the hydrolethalus syndrome protein HYLS-1 as a protein required for TF assembly in *C. elegans*. In addition to TF disorganization, *hyls-1* mutants display defects in docking and entry of IFT particles, TZ organization, axoneme assembly and ciliary gating. Intriguingly, co-deletion of the TF component DYF-19 further exacerbates TZ anomalies and completely abrogates ciliogenesis, supporting a role for TF-mediated selective gating in cilia assembly.

## Results

### HYLS-1 regulates TF assembly

**Figure 1:**
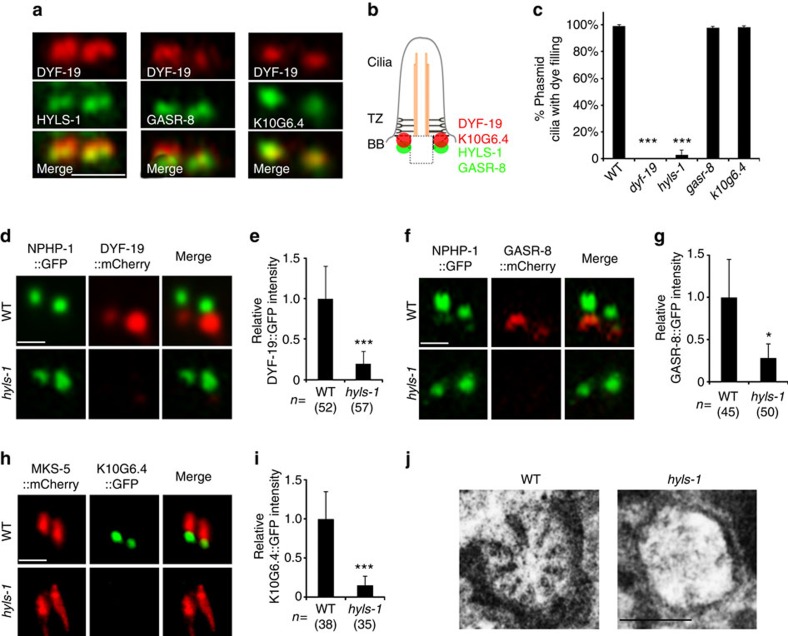
HYLS-1 promotes TF assembly. (**a**) Localization of DYF-19, HYLS-1, GASR-8 and K10G6.4 in tail phasmid cilia. Each phasmid organ contains two cilia bundled together^40^. Images show the ciliary base of one set of two phasmid cilia expressing mCherry-tagged DYF-19 and GFP-tagged candidate proteins as indicated. HYLS-1 and GASR-8 localize proximal to DYF-19 in the region of the degenerated basal body, whereas K10G6.4 completely co-localizes with DYF-19 on TFs. (**b**) Cartoon illustrating the relative localization of DYF-19, HYLS-1, GASR-8 and K10G6.4 at the ciliary base. (**c**) Dye-filling of phasmid cilia was used to analyse cilia integrity. *hyls-1* and *dyf-19* mutants are dye-fill defective, whereas *gasr-8* and *k10g6.4* mutants possess apparently normal cilia. More than 200 worms analysed for each genetic background. (**d**–**i**) HYLS-1 is required for proper localization of DYF-19, GASR-8 and K10G6.4. Phasmid cilia expressing fluorescently tagged DYF-19 (**d**), GASR-8 (**f**) and K10G6.4 (**h**) in WT and *hyls-1* mutants. NPHP-1 (**d**,**f**) and MKS-5 (**h**) were used to label the TZ. Quantification of relative fluorescence intensities of DYF-19 (**e**), GASR-8 (**g**) and K10G6.4 (**i**) in cilia of WT and *hyls-1* mutants. *n* represents number of cilia analysed. (**j**) BB remnant and TFs are missing at the base of *hyls-1* amphid cilia. Scalebars, 200 nm (**j**), 1 μm (other panels). Error bars indicate s.d. Student's *t*-test indicates significant differences; *P<0.01 and ***P<0.001.

We previously identified DYF-19 (the orthologue of human FBF1) as the first genuine TF component in *C. elegans*^17^. Loss of DYF-19 compromises the entry of IFT particles, suggesting that TFs may be a critical site for the docking and sorting of IFT particles^17^. However, the fact that DYF-19-depleted worm cilia still possess TFs indicated that the key factors regulating TF biogenesis remain to be identified. To further understand the role of TFs in the context of cilia, we sought to identify additional TF components. Using mCherry-tagged DYF-19 as a TF marker, we examined the localization of green fluorescent protein (GFP)-tagged versions of known or predicted ciliary proteins in *C. elegans*^31^,^32^. This search led us to identify three TF-associated components, GASR-8, the putative homologue of human GAS8, and K10G6.4, related to human ANKRD26, as well as HYLS-1, a conserved centriolar and basal body component^33^. Among them, HYLS-1 and GASR-8 localize proximal to DYF-19, suggesting they may be proteins associated with or closely adjacent to TFs, whereas K10G6.4 completely co-localizes with DYF-19 on TFs (Fig. 1a,b). GAS8 dysfunction causes primary ciliary dyskinesia with mild axonemal disorganization in humans and disrupts hedgehog signalling in zebrafish. However, the underlying molecular mechanisms remain poorly defined^34^,^35^,^36^. Similarly, *ANKRD26* mice displayed obesity phenotypes potentially linked to defects in primary cilia^37^. Finally, mutations in HYLS1 have been detected in individuals with hydrolethalus syndrome^38^, a putative ciliopathy, and HYLS1 and its worm homologue HYLS-1 have been reported to be required for ciliogenesis in vertebrates and *C. elegans*, respectively^33^.

To investigate the role of the three proteins, we obtained loss-of-function mutants of *hyls-1 (tm3067)*, *gasr-8 (gk1232)* and *k10g6.4 (gk567)*. All three mutants carry large deletions in their coding region and are putative *null* alleles (Supplementary Fig 1a)^33^. In *C. elegans*, the head amphid and tail phasmid cilia take up lipophilic fluorescent dye from the external environment^39^. Animals with abnormal ciliogenesis fail to take up dye and are considered dye-filling defective (Dyf)^40^. We found that, similar to *dyf-19* mutants, *hyls-1* mutants showed severe dye-filling defects as previously reported^33^, whereas *gasr-8* and *k10g6.4* mutants appeared to possess normal cilia (>200 animals analysed for each mutant; Fig. 1c). Remarkably, GFP-tagged DYF-19, GASR-8 and K10G6.4, all lost their ciliary targeting in *hyls-1* mutants (Fig. 1d–i). In contrast, loss of GASR-8, K10G6.4 or DYF-19 did not affect the localization of other TF components (data not shown). These data suggest that, among identified TF or TF-associated components in *C. elegans*, HYLS-1 may be the key factor required for TF organization.

We further used serial-section transmission electron microscopy (TEM) to examine ciliary ultrastructure. The centriole-derived basal body is a near-invariant feature at the base of the cilium in most species. However, it is not so in *C. elegans*, where basal bodies largely degenerate during development via mechanisms that are currently unclear^33^,^40^. The only remaining basal body structures have been reported to be dense fibrous TFs^40^. Of note, recent cryo-tomography studies suggest that some of the electron densities observed at the ciliary base in conventional TEM studies are the result of two-dimensional projections of microtubule flares^41^, which may be the remnant of the degenerated basal body. Interestingly, this remnant as well as the fibrous structures universally observed at the ciliary base in wild type (WT) was missing or highly aberrant in *hyls-1* cilia (Fig. 1j and Supplementary Table 1). This probably explains why DYF-19, GASR-8 and K10G6.4 display impaired targeting in *hyls-1* mutants.

### HYLS-1 is required for IFT recruitment and entry

**Figure 2:**
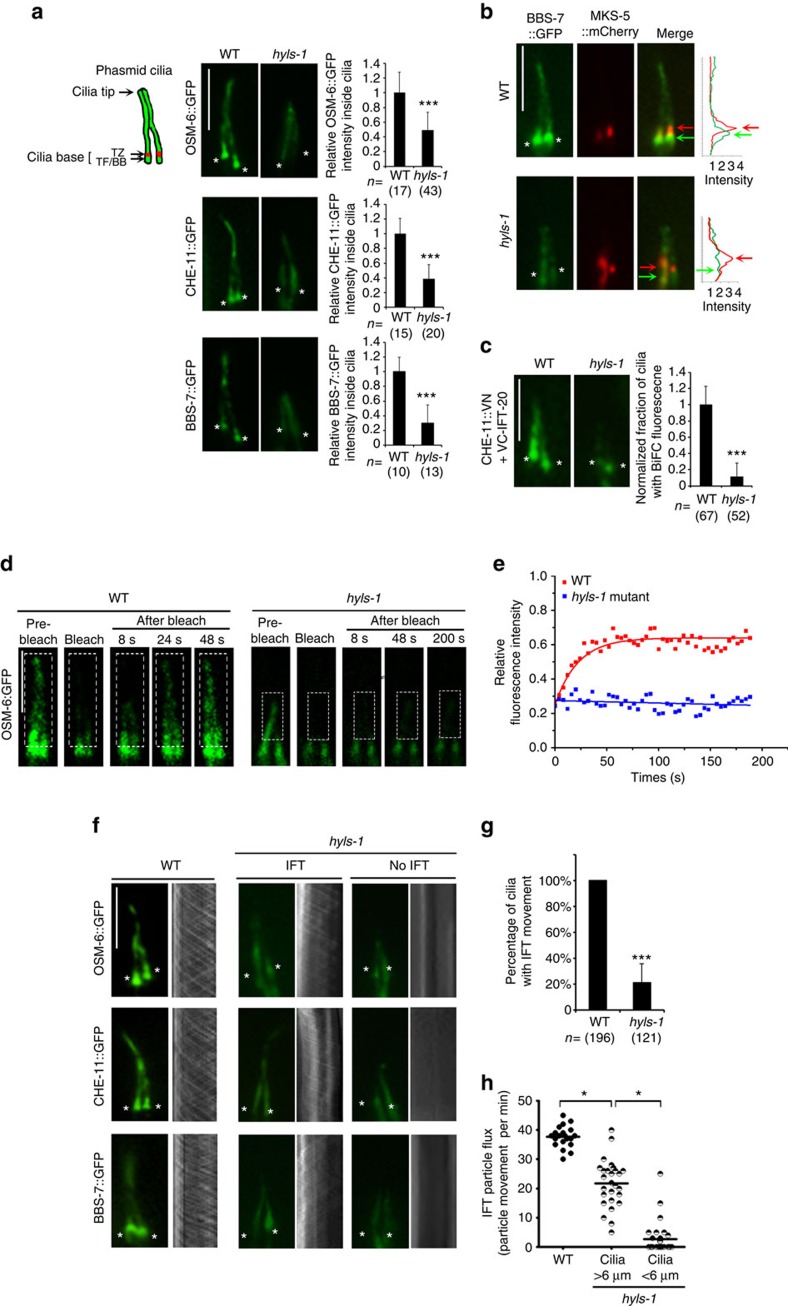
HYLS-1 is required for IFT recruitment and ciliary entry. (**a**) Localization of IFT components in WT and *hyls-1* mutant phasmids. Schematic of phasmid sensory organ. Each phasmid contains two cilia whose distal segments bundle together. Image panels show phasmid cilia in WT and *hyls-1* mutants expressing GFP-tagged IFT components (OSM-6, the orthologue of IFT-B component IFT52; CHE-11, the orthologue of IFT-A component IFT140; BBS-7, the orthologue of BBSome component BBS7) alongside quantification of their fluorescence intensities inside the cilium proper. IFT signal is significantly reduced in *hyls-1* mutants compared with that in WT. In contrast to the strong accumulation in WT, *hyls-1* mutants further show no enrichment of IFT components at the ciliary base. Asterisks indicate cilia base. *n* represents number of cilia analysed. (**b**) Representative images of WT and *hyls-1* cilia co-labelled with GFP-tagged BBS-7 and mCherry-tagged TZ marker MKS-5. BBS-7 strongly accumulates below the TZ in WT but not *hyls-1* mutants. Corresponding fluorescence intensities are shown in right panels. Asterisks indicate cilia base. Red arrows indicate TZ, green arrows indicate TFs/BB. (**c**) BiFC reveals association between IFT-A and IFT-B components in both WT and *hyls-1* mutant phasmids. However, complementation is restricted to the ciliary base in *hyls-1* mutants as revealed by the quantification of BiFC signal within cilia (right panel). *n* represents number of cilia analysed. (**d**,**e**) Fluorescence recovery after photobleaching shows that ciliary entry of IFT is severely disrupted in *hyls-1* mutants. (**d**) Representative images before/after photobleaching (boxed region) of GFP-tagged OSM-6 signal in WT and *hyls-1* mutants. (**e**) Quantification and curve fit for experiment shown in **d**. (**f**) Representative images and corresponding kymographs illustrating GFP-tagged IFT particle movement. In *hyls-1* mutants, IFT movement is either weak in cilia or non-detectable. (**g**) Only ∼20% *hyls-1* cilia display detectable IFT movement. *n* represents number of cilia analysed. (**h**) Quantification reveals a significant reduction in anterograde IFT particle flux in *hyls-1* mutants compared with that in WT. Each data point represents a single measurement. Scale bars, 5 μm. Error bars indicate s.d. Significant differences identified by Student's *t*-test; *P<0.01 and ***P<0.001.

To further assess the functional consequences of HYLS-1 deletion, we introduced various IFT markers into *hyls-1* mutants. Surprisingly, we found that truncated cilia still form in ∼80% of *hyls-1* mutants (Fig. 2a), suggesting that the early steps of ciliogenesis, including basal body-to-membrane docking, are largely unaffected. However, fluorescence intensity analysis indicates that the ciliary levels of all IFT components examined, including the IFT-A component CHE-11, the IFT-B component OSM-6 and the BBSome component BBS-7, are decreased by 50–75% in *hyls-1* mutants compared with WT animals (Fig. 2a). Total protein levels as well as fluorescence intensities in the cell body are unchanged (Supplementary Fig. 2a–e), indicating that loss of HYLS-1 does not affect expression or stability of IFT proteins. IFT components normally show strong accumulation in the vicinity of TFs in WT worms (Fig. 2a,b). These accumulations do not occur in *hyls-1* mutants (Fig. 2a,b). We previously reported that the TF component DYF-19 recruits and facilitates ciliary docking/entry of IFT particles^17^. To examine how loss of HYLS-1 affects IFT particles, we used bimolecular fluorescence complementation (BiFC) to analyse the assembly and transport of IFT particles by visualizing the *in vivo* association between IFT components in the worm^17^,^42^. As shown in Fig. 2c, the IFT-B component IFT-20 shows strong fluorescence complementation with the IFT-A component CHE-11, the Kinesin-2 subunit KAP-1 and the dynein subunit XBX-1 in both WT or *hyls-1* mutants, indicating that assembly of the IFT-A–IFT-B complex, as well as the association of IFT particles with motors, is largely normal in *hyls-1* cilia (Fig. 2c and Supplementary Fig. 2a–c). However, most fluorescence complementation signal of BiFC pairs was restricted to the ciliary base in *hyls-1* mutants, suggesting that assembled IFT particles fail to efficiently enter cilia in *hyls-1* cilia (Fig. 2c).

We further performed fluorescence recovery after photobleaching (FRAP) to obtain live cell imaging evidence that the ciliary entry of IFT is compromised in *hyls-1* cilia. FRAP analysis was performed on phasmid cilia, bleaching the cilium proper and examining recovery of GFP signal from the cytoplasm. In WT, ∼60% of the signal for the GFP-tagged IFT-B component OSM-6 recovers with a half time (*t*_1/2_) of 23.7±3.2 s (data represented as ±s.d.), as determined from a single exponential fit to the data (Fig. 2d,e). By contrast, no detectable recovery of OSM-6::GFP fluorescence was recorded over 3 min in *hyls-1* cilia (Fig. 2d,e), supporting the conclusion from BiFC experiments that the ciliary entry of IFT machinery is severely disrupted in *hyls-1* cilia.

We next directly analysed IFT transport. Interestingly, although *hyls-1* mutants displayed consistent albeit reduced cilia signal for individual IFT components, only ∼20% of *hyls-1* cilia showed active IFT particle movement in time-lapse imaging (Fig. 2f,g). This is not simply due to reduced IFT protein levels, because, first, our camera is sensitive enough to detect IFT movement in cilia with even weaker IFT signal and, second, even *hyls-1* cilia with higher residual signal frequently showed no IFT movement at all. In addition, in *hyls-1* cilia with detectable IFT movement, the frequency of IFT transport was significantly lower (Fig. 2g). These results further confirm the defective ciliary entry of IFT machinery in *hyls-1* mutants and also suggest that individual IFT components leak into cilia without being incorporated into functional IFT particles. Interestingly, we found that reduced frequency of IFT transport correlates with defects in cilia elongation (Fig. 2h). We thus conclude that, in *hyls-1* cilia, impaired entry of functional IFT complexes results in defective ciliogenesis.

### Deletion of DYF-19 in *hyls-1* mutants abrogates ciliogenesis

**Figure 3:**
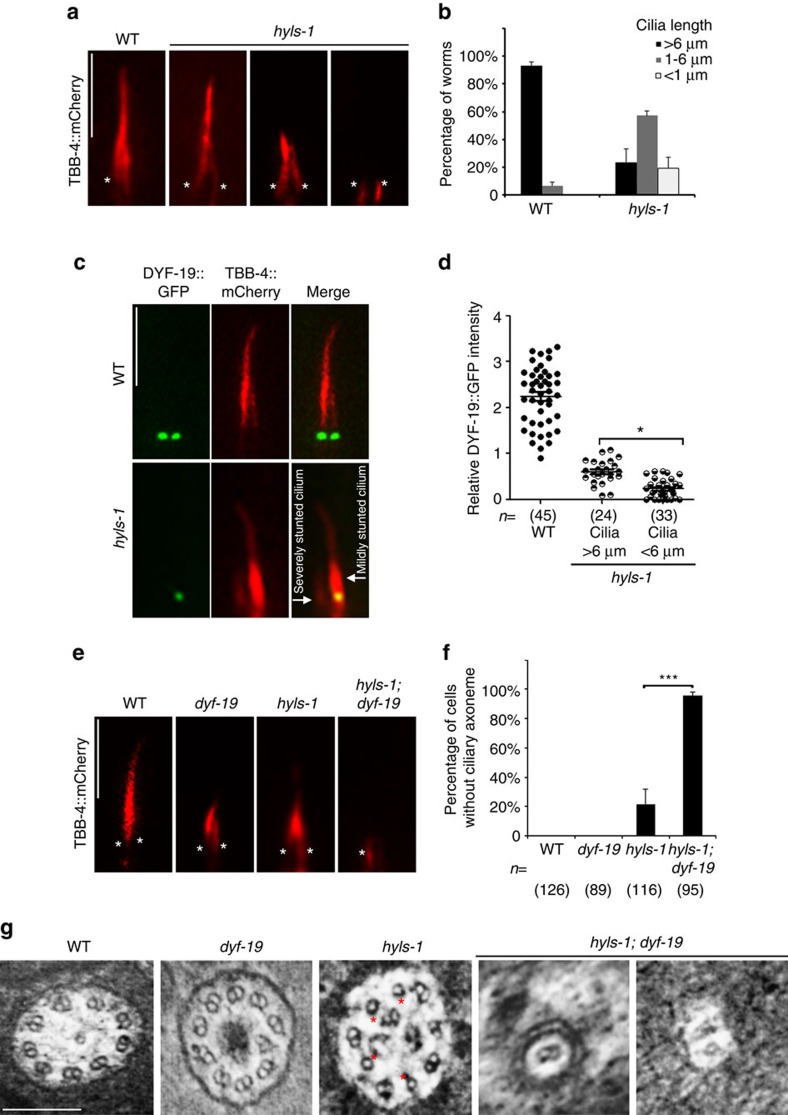
Additional deletion of DYF-19 in *hyls-1* mutants completely abrogates ciliogenesis. (**a**) *hyls-1* mutants exhibit variable defects in axoneme elongation. Phasmid cilia were labelled with the mCherry-tagged axoneme marker β-tubulin TBB-4. Asterisks indicate cilia base. (**b**) Quantification of cilia length in WT and *hyls-1* mutants. Average of three independent experiments. *n*>100 cilia for each genetic background in each experiment. (**c**) Representative images of phasmid cilia co-labelled with mCherry-tagged TBB-4 and GFP-tagged DYF-19. The severity of cilia truncation in *hyls-1* mutants is correlated with the level of residual DYF-19 at the cilia base. (**d**) Quantification of DYF-19 signal in WT and *hyls-1* mutants. *n* represents the number of cilia analysed. (**e**) Additional deletion of DYF-19 in *hyls-1* mutants completely abrogates ciliogenesis. Cilia are labelled with TBB-4. Asterisks indicate cilia base. (**f**) Quantification of phasmid neurons without visible axoneme based on OSM-6::GFP in WT, *hyls-1*, *dyf-19* and *hyls-1; dyf-19* double mutants, respectively. *n* represents the number of phasmid neurons analysed. (**g**) TEM analysis of the proximal axoneme (immediately distal to the TZ) of amphid neurons. Compared with WT and *dyf-19* axonemes that consist of nine doublet microtubules, *hyls-1* mutants frequently display missing B-tubules (red stars). *dyf-19; hyls-1* double mutants show severely compromised and stunted axonemes that possess only one or two microtubules and immediately terminate only one TEM section (∼80 nm) beyond the TZ. Scale bars, 200 nm (**g**), 5 μm (**a**,**c**,**e**). Error bars indicate s.d. Student's *t*-test indicates significant differences; *P<0.01 and ***P<0.001.

The ciliogenesis defect in *hyls-1* mutants is variable, with cilia lengths ranging from mildly reduced to severely stunted. When visualized with mCherry-tagged β-tubulin TBB-4 as an axonemal marker, >98% of WT cilia are longer than 6 μm. By contrast, ∼60% of *hyls-1* cilia are between 1 and 6 μm in length and ∼20% are below 1 μm in length or are absent (Fig. 3a,b). We previously noted that, despite severe TF defects, ∼10% DYF-19 still localizes to the ciliary base in *hyls-1* mutants (Fig. 1e). As DYF-19/FBF1 is a key TF component promoting the ciliary entry of IFT complexes^17^, we hypothesized that the remaining DYF-19 could facilitate residual IFT entry and contribute to ciliogenesis in *hyls-1* mutants. Consistent with this, in *hyls-1* mutant worms co-expressing mCherry-tagged TBB-4 and GFP-tagged DYF-19, DYF-19 signal was indeed stronger in mildly truncated cilia than in severely stunted cilia (Fig. 3c,d), indicating that the level of the remaining DYF-19 at the ciliary base correlates with the severity of ciliogenesis defect.

To further test our hypothesis that residual DYF-19 at the ciliary base supports incomplete ciliogenesis in *hyls-1* mutants, we constructed *hyls-1; dyf-19* double mutants. Strikingly, double mutants showed no cilia at all (Fig. 3e,f). TEM analysis showed that the axoneme terminates immediately distal to the TZ with only a few microtubules observed in *hyls-1; dyf-19* double mutants, indicating that axoneme elongation is almost completely blocked (Fig. 3g and Supplementary Fig. 3a). We did not observe any genetic interactions between *hyls-1* and *gasr-8* or *k10g6.4* (Supplementary Fig. 3b,c). Based on these observations we propose that, in the absence of HYLS-1, TFs are disorganized but residual DYF-19 at the ciliary base still supports partial ciliogenesis by recruiting and facilitating the ciliary entry of IFT particles. Additional deletion of DYF-19 in TF-deficient cilia that result from *hyls-1* knockout completely abrogates ciliogenesis.

### Loss of HYLS-1 disrupts TZ integrity and function

**Figure 4:**
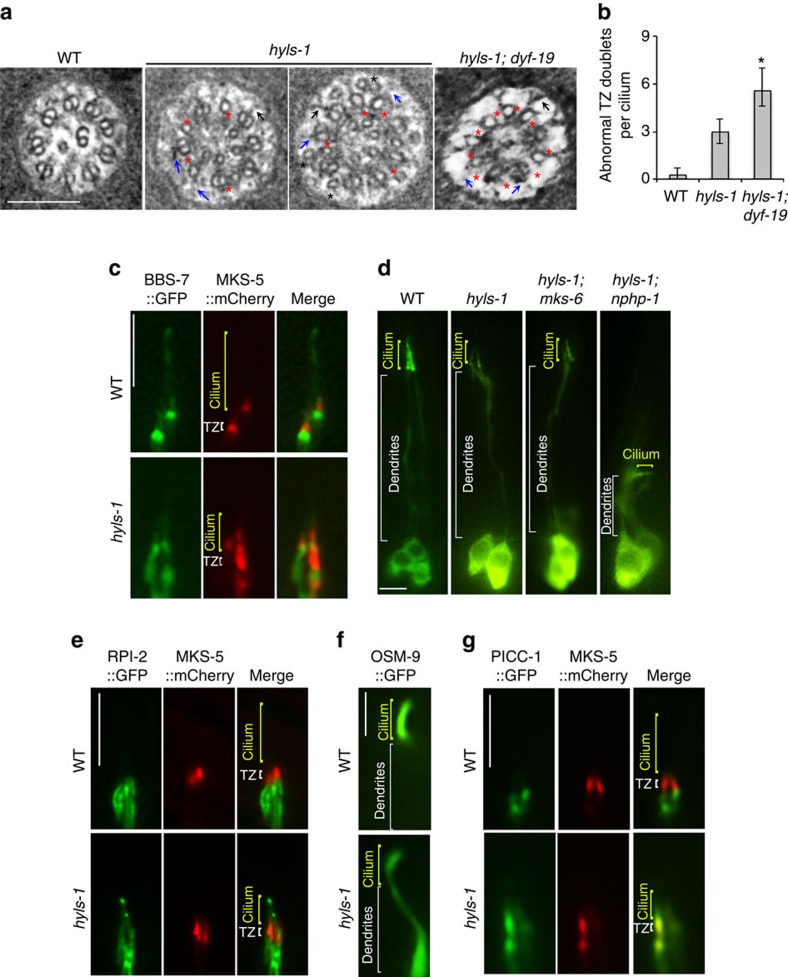
HYLS-1 is required for TZ integrity and function. (**a**) TEM analysis of the TZ. *hyls-1* mutants display a range of anomalies in the TZ, including missing B-tubules (red stars), putative broken Y-links (blue arrows) and displaced singlet microtubules (black stars). Some Y-links still form and associate with the membrane (black arrows). *hyls-1; dyf-19* double mutants display more severe defects with an increased number of incomplete doublet microtubules. (**b**) Quantification of incomplete doublet microtubules in the TZ in WT, *hyls-1* and *hyls-1; dyf-19* double mutants. See Supplementary Table 1 for the numbers of cilia analysed for each genetic background. Error bars indicate s.d. Student's *t*-test for significance; *P<0.01. (**c**) Fluorescence images of phasmid cilia co-expressing GFP-tagged BBS-7 and mCherry-tagged MKS-5 in WT and *hyls-1* mutants. MKS-5 signal exclusively enriches around the TZ in WT cilia, but diffuses to a larger area including the cilia proper in *hyls-1* mutants. (**d**) Dendrite extension is compromised in *hyls-1; nphp-1* but not *hyls-1; mks-6* double mutants. Representative images of dendrites visualized by expression of GFP-tagged IFT-B component OSM-6 illustrate genetic interaction between HYLS-1 and TZ proteins. See also Supplementary Fig. 4b. (**e**–**g**) Ciliary gating for both membrane-associated and soluble proteins is perturbed in *hyls-*1 mutants. Representative images of phasmid cilia co-expressing GFP-tagged proteins and mCherry-tagged MKS-5 as a TZ marker. (**e**) The non-ciliary membrane protein RPI-2 is restricted to below the TZ in WT but abnormally enters phasmid cilia in *hyls-1* mutants. (**f**) OSM-9, a membrane receptor, enriches in the cilia of OLQ (outer labial quadrant) neurons in WT worms, but mislocalizes along dendrites in *hyls-1* mutants. (**g**) The non-ciliary cytoplasmic protein PICC-1 is restricted below the TZ in WT but abnormally enters phasmid cilia in *hyls-1* mutants. Scale bars, 200 nm (**a**), 5 μm (**c**–**g**).

Although HYLS-1 does not localize to the TZ, an unexpected discovery was that *hyls-1* mutants exhibit profound irregularities in the TZ: the B-tubules of the outer microtubule doublets are frequently missing and some Y-links, especially those on incomplete doublets, are missing or not intact (Fig. 4a). These axonemal anomalies are unique and distinct from those of known TZ mutants and in other ciliogenesis mutants in *C. elegans*, in which the doublet microtubules of the TZ are usually unaffected^11^,^40^,^43^. Despite the severe disruption of TZ architecture, most TZ components examined, including MKS-3, MKS-6, MKSR-2, NPHP-4 and CCEP-290, show normal targeting in *hyls-1* mutants (Supplementary Fig. 4). However, MKS-5, which is proposed to be the central component required for TZ formation^43^,^44^,^45^, is mislocalized both above and below the TZ in *hyls-1* mutants (Fig. 4c).

Given that HYLS-1 was first identified as a centriolar protein interacting with SAS-4 (ref. 33), one explanation is that TZ anomalies stem from impaired templating of axonemal microtubules by the basal body. Alternatively, defective TF formation might cause deregulated cilia entry of key factors required for axonemal microtubule formation and/or stability. To distinguish between these possibilities, we examined the TZ in *hyls-1; dyf-19* double mutants. Strikingly, more B-tubules are missing in the TZ of *hyls-1; dyf-19* cilia compared with *hyls-1* single mutants (Fig. 4a,b). DYF-19 is a specific TF component and does not directly affect TZ organization and axonemal microtubules^17^. We reason that depletion of DYF-19 in *hyls-1* mutants further impairs TF function, exacerbating impaired ciliary entry of key proteins required for TZ organization. We thus propose that TZ defects in *hyls-1* mutants are at least in part due to defects in TF function.

TZs have been linked to cell-matrix anchorage/adhesion during dendrite extension, with loss of multiple TZ modules resulting in drastically shortened dendrites^11^,^43^,^46^,^47^. Except for *mks-5*, mutations in *mks* genes (*mks-1*, *mks-2*, *mks-3*, *mks-6*, *mksr-1* and *mksr-2*) or *ccep-290* do not affect dendrite extension unless combined with mutations in *nphp* genes (*nphp-1* and *nphp-4*), conditions which result in severely disorganized TZs and dendrite collapse^11^,^43^,^46^,^47^. Interestingly, we found that dendrite collapse could be observed in *hyls-1; nphp-1* and *hyls-1; mks-5* double mutants, but not in *hyls-1* single mutants or *hyls-1; mks-1* ans *hyls-1; mks-3*, or *hyls-1; mks-6* double mutants (Fig. 4d and Supplementary Fig. 4b). The synergistic defect in dendrite extension in *hyls-1; nphp-1* and *hyls-1; mks-5* double mutants may be caused by aggravated TZ disorganization, as depletion of HYLS-1 alone is sufficient to cause MKS-5 mislocalization and TZ anomalies. Alternatively, TFs may directly contribute to cell-matrix adhesion.

The TZ is known to form part of the ciliary gate restricting entry of non-ciliary membrane-associated proteins^43^. We therefore tested whether deletion of HYLS-1 also affects ciliary gating. RPI-2, the *C. elegans* orthologue of human X-linked retinitis pigmentosa 2, which associates with the plasma membrane in sensory neurons, and the transmembrane protein TRAM-1 are excluded from the ciliary membrane in WT but abnormally leak into cilia in TZ mutants^17^,^43^. As shown in Fig. 4e and Supplementary Fig. 4c, we observed abnormal ciliary entry of both RPI-2 and TRAM-1 in *hyls-1* cilia, indicating that the diffusion barrier for membrane proteins is compromised in HYLS-1-deficient cilia. We further examined the localization of the ciliary sensory receptor OSM-9, which specifically targets to OLQ neuronal cilia in WT worms. This ciliary enrichment is disrupted in *hyls-1* mutants, with a strong mislocalization to below the ciliary base (Fig. 4f). A compromised diffusion barrier at cilia base could lead to the lateral leak (diffusion) of proteins that should be enriched inside the cilia. An alternative explanation is that selective import of cilia-specific sensory receptors is disrupted in *hyls-1* cilia. We next investigated whether gating for soluble proteins is also affected in *hyls-1* mutants. PICC-1, the orthologue of human CCDC85A, and DAF-21, the orthologue of HSP90, do not enter cilia in WT animals (Fig. 4g and Supplementary Fig. 4d). In contrast, significant amounts of both proteins gain access to the cilia lumen in *hyls-1* mutants (Fig. 4g and Supplementary Fig. 4d), indicating that loss of HYLS-1 also disrupts ciliary gating for soluble proteins. Collectively, these results indicate that HYLS-1 is essential for gating of both membrane and soluble proteins by regulating the proper architecture of TFs and/or TZ.

## Discussion

**Figure 5:**
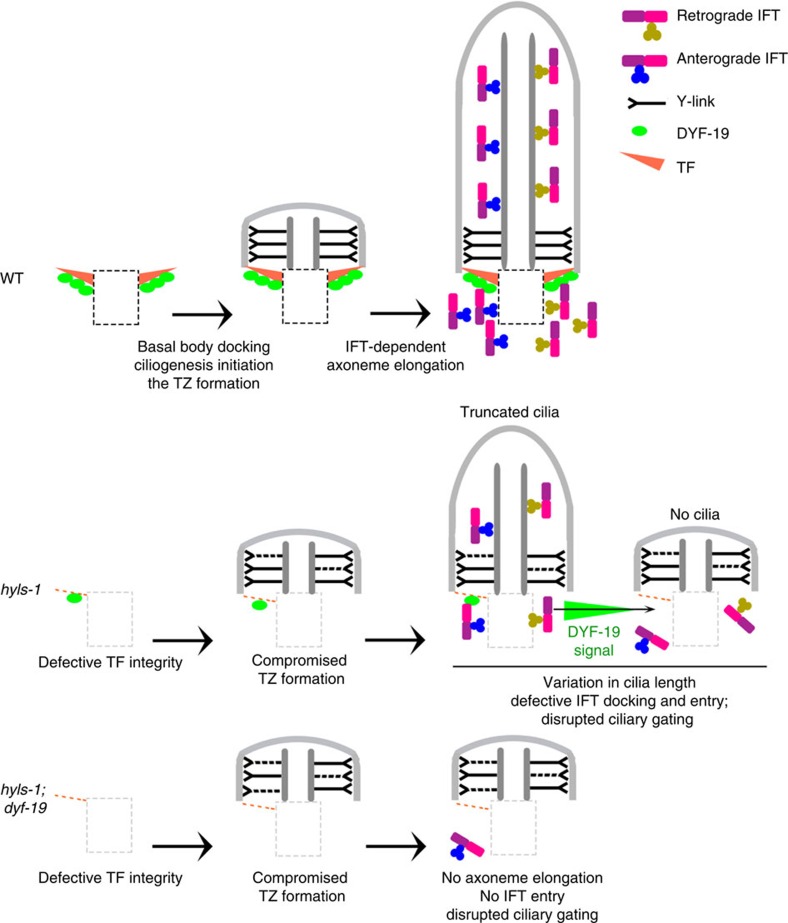
Working model of HYLS-1 function in ciliogenesis. Ciliogenesis involves a hierarchy of steps: TF maturation, basal body docking, TZ formation, IFT anchoring and entry and axoneme elongation. TFs and the TZ are proposed as important parts of the ciliary gate. HYLS-1 is essential for basal body stability and TF assembly. *hyls-1* mutants also show defects in TZ integrity, compromised cilia gating and truncated axonemes. The formation of truncated cilia in *hyls-1* mutants correlates with residual targeting of the TF component DYF-19 to the ciliary base. Depletion of DYF-19 in *hyls-1* mutants abrogates ciliogenesis, potentially by abolishing IFT entry. TZ structural anomalies are also exacerbated in *hyls-1; dyf-19* double mutants, suggesting that TFs contribute to ciliary entry of proteins required for TZ integrity.

Taken together, we found that HYLS-1 is required for formation of the ciliary gate. In *hyls-1* cilia, the docking/import of IFT machinery and the formation of the TZ and axoneme are compromised (Fig. 5). In addition, ciliary gating for both membrane and soluble proteins is disrupted, resulting in mis-localization of both ciliary and non-ciliary proteins. Interestingly, residual amount of the TF component DYF-19 continue to target to *hyls-1* cilia and facilitate minimal IFT entry and axonemal elongation. Co-deletion of DYF-19 in *hyls-1* cilia further exacerbates TZ malformation and totally abrogates ciliogenesis (Fig. 5). Although loss of TFs and TF-associated proteins has previously been reported to impair recruitment of ciliary proteins in vertebrates^16^,^19^,^22^,^23^,^24^, the interpretation of these results is complicated by the failure of basal bodies to dock to the plasma membrane, a defect not observed in *C. elegans hyls-1* mutants. Our findings therefore strongly support the proposed role for TFs in ciliary import^17^.

Recent cryo-tomography analysis suggests that the TF-like structures observed by conventional TEM in *C. elegans*, at least in part, are two-dimensional projected images of microtubule flares at the ciliary base^41^, which may be the remnant of the degenerated basal body. The apparent loss of these flares in *hyls-1* mutants suggest additional basal body defects, which we are currently investigating. Nevertheless, the presence of fibrous connections between those microtubules and the plasma membrane (see Fig. 4c and Supplementary Fig. 2c), and the localization of DAF-19/FBF1, a *bona fide* TF component in vertebrates^17^, to precisely this region support the conservation of key aspects of TF structure in worms. It will be interesting to determine the extent of this conservation and relate it to the three-dimensional architecture of TFs in worms and other species.

HYLS-1 is unlikely to be a structural component of TFs based on the fact that it was identified as a core centriolar component^33^ and only partially co-localizes with TF components in *C. elegans* (Fig. 1a). Instead, we speculate that HYLS-1 may regulate TF assembly by conditioning the distal end of the centriole to enable structural components to dock, to form TFs. Currently, four other ciliopathy associated proteins, OFD1, ODF2, C2CD3 (C2 calcium-dependent domain containing 3) and DZIP1 (DAZ-interacting zinc finger protein 1), have been implicated in TF formation by acting on the outer wall of the mother centriole to promote fibre/appendage assembly^24^,^28^,^48^,^49^,^50^. It will be interesting to investigate the functional relationship between HYLS1 and these proteins. Intriguingly, HYLS-1 is the only protein besides C2CD3 to have a homologue in the *C. elegans* genome, suggesting that it may be a key factor in regulating TF formation across species^33^,^51^.

In sum, we provide mechanistic insights into how the ciliopathy protein HYLS-1 contributes to ciliogenesis and establishment of the ciliary gate. In *C. elegans*, the TZ is required for gating of membrane proteins but dispensable for IFT-dependent axoneme assembly^11^,^43^,^46^,^47^. In contrast, loss of HYLS-1 compromises ciliogenesis, severely disrupts TZ architecture and affects gating for both membrane and soluble proteins. These defects appear to stem at least in part from a failure of selective entry of ciliary components including the IFT machinery through TFs. Therefore, the TZ and TFs appear to serve distinct functions in ciliary gating, with the TZ acting primarily as a passive barrier to ciliary entry, whereas TFs help to actively load components enriched in the ciliary compartment.

## Methods

### Strains

Worms were cultured, maintained and crossed using standard procedures^52^. All *C. elegans* strains and reporters used in this study are listed in Supplementary Table 2.

### Dye-filling assays

Worms were incubated in 1% DiI (Molecular Probes) in M9 buffer for 2 h at room temperature and allowed to destain for ∼30 min on a seeded Nematode Growth Medium (NGM) plate before analysis. Cell bodies with positive dye filling in phasmid cilia were scored under a Nikon TE 2000-U microscope with a Plan Apochromat × 60, 1.49 numerical aperture (NA) oil objective.

### Microscopy and imaging

Young adult worms were mounted on a 5% agarose pad, immobilized with 10 mM levamisole and imaged using a Nikon TE 2000-U microscope (for IFT images) or Zeiss LSM 780 confocal laser-scanning microscope (for images in Fig. 1a). IFT movement was examined on the Nikon TE 2000-U microscope using a Plan Apochromat × 100, 1.49 NA oil objective and Photometrics QuantEM 512SC charge-coupled device camera (Roper Scientific). For IFT analysis, time-lapse sequences were acquired at 200 ms intervals for either WT or *hyls-1* cilia. Metamorph software (Molecular Devices) was then used to trace the movement of GFP-tagged IFT particles in either amphid or phasmid cilia. Kymographs were generated from time-lapsed image stacks by using the kymograph plugin in Metamorph on regions of interest encompassing the entire cilium and applying a maximum intensity projection to display the change in fluorescence distribution along cilia over time. The velocity of IFT movement was calculated in Metamorph from the trajectory path of IFT particles in the kymograph plot. The number of IFT particles per cilium per min, referred to as IFT particle flux, was also determined from these plots.

### BiFC assay

The Split-Venus BiFC assay was used to examine IFT-A and IFT-B complex formation and motor association in living worms^53^. For this, complementary DNAs of relevant genes were inserted into the BiFC vectors VN173 and VC155 under the control of the ciliated-cell-specific promoter of *arl-13* and injected at 5 ng μl^−1^ into WT worms together with the co-injection marker pRF4 (rol-6(su1006)). The following BiFC pairs were used in this study: the IFT-A component CHE-11::VN173 and the IFT-B component IFT-20::VC155; the IFT component IFT-20::VC155 and the dynein component XBX-1::VN173; and the IFT component IFT-20::VC155 and the kinesin II motor component KAP-1::VN173. Fluorescent signals were visualized using the YFP filter under a Nikon TE 2000-U microscope.

### Fluorescence intensity measurement

Identical exposure conditions were used for samples from the same experiment. Quantification of fluorescence intensities was performed using Nikon's NIS-Elements microscope imaging software. Cilia signal was determined using the manual measurement tool, drawing an area around the cilia proper, excluding the cilia base. Background signal was measured in the area surrounding the cilium and subtracted. The average value of signals in WT was normalized to one.

### Fluorescence recovery after photobleaching

FRAP assays were carried out on a Zeiss LSM 510 confocal microscope with a × 100, 1.46 NA oil objective at 25 °C. A 488 nm laser (25 mW) at 80% power was used for photobleaching and images acquired every 4 s. All images were acquired using the same settings. After background subtraction, the data were normalized to the pre-bleach fluorescence. The recovery curve was fitted to a single-exponential equation *F*(*t*)=*F*_0_+(*F*_inf_−*F*_0_)(1−*e*^−*kt*^), where *F*(*t*) is the total fluorescence at time *t* after the bleach, *k* is the constant describing the rate of recovery, *F*_0_ is the fluorescence immediately post bleach (0 min time point) and *F*_inf_ is the maximum recovered fluorescence. The recovery half-time was calculated by *t*_1/2_=ln 2/*k*.

### Immunoprecipitation and western blotting

WT and *hyls-1* mutant worms expressing OSM-6::GFP were washed off twenty 10 cm plates and transferred into an Eppendorf tube filled with M9 buffer with 0.1% Triton X-100, pelleted at 100–200 g for 3 min, washed 3 times with 1 ml M9 with 0.1% Triton, then lysed in binding buffer (25 mM Tris.Cl pH 7.6, 150 mM NaCl, 1 mM EDTA and 0.5% Triton X-100) by grinding in liquid nitrogen. Lysate (1/10) was kept to load on gel to blot for β-actin. OSM-6::GFP was immunoprecipitated from the remaining 9/10 of the lysate with anti-GFP monoclonal antibody (A-11120, Invitrogen) then incubated with 30 μl protein G Sepharose beads in binding buffer with Complete Protease Inhibitor Cocktail (Roche) overnight at 4 °C. After washing three times with binding buffer, about half of immunoprecipitates was loaded on a 7.5% SDS–PAGE gel and immunoblotted. The primary antibodies used were mouse monoclonal anti-GFP (A-11120, Invitrogen, diluted 1:500) and β-actin antibody (C4) (sc-47778, Santa Cruz, 1:5,000) as a loading control. Proteins were detected using secondary antibodies conjugated to horseradish peroxidase (1:2,000, Jackson Immunoresearch) and SuperSignal West Femto Luminol Enhanced ECL detection kit (Thermo Scientific). Visualization of protein bands was performed using the ChemiDoc XRS imaging system (Bio-Rad). Band intensities were measured using ImageJ (National Institutes of Health).

### Transmission electron microscopy

TEM on amphid channel cilia was performed using standard procedures by the EM core facility at the Mayo Clinic. Worm heads were fixed in 2.5% glutaraldehyde in cacodylate buffer and postfixed in 1% osmium tetroxide in cacodylate buffer. Samples were dehydrated and embedded in Embed812 resin according to standard procedures^53^. Serial sections (∼80 nm thickness) from the anterior tip of worm head were collected and viewed on an electron microscope (JEM-1400; JEOL). More than ten worms were cut for each strain.

### Data availability

All relevant data supporting the findings of this study are either included within the article and its Supplementary Information files or available upon request from the corresponding author.

## Additional information

**How to cite this article**: Wei, Q. *et al*. The hydrolethalus syndrome protein HYLS-1 regulates formation of the ciliary gate. *Nat. Commun.* 7:12437 doi: 10.1038/ncomms12437 (2016).

## Supplementary Material

Supplementary Information Supplementary Figures 1-4, Supplementary Tables 1 & 2

## References

[b1] Singla V. & Reiter J. F. The primary cilium as the cell's antenna: signalling at a sensory organelle. Science 313, 629–633 (2006). 16888132 10.1126/science.1124534

[b2] Goetz S. C. & Anderson K. V. The primary cilium: a signalling centre during vertebrate development. Nat. Rev. Genet. 11, 331–344 (2010). 20395968 10.1038/nrg2774 PMC3121168

[b3] Badano J. L., Mitsuma N., Beales P. L. & Katsanis N. The ciliopathies: an emerging class of human genetic disorders. Annu. Rev. Genomics Hum. Genet. 7, 125–148 (2006). 16722803 10.1146/annurev.genom.7.080505.115610

[b4] Hildebrandt F., Benzing T. & Katsanis N. Ciliopathies. N. Engl. J. Med. 364, 1533–1543 (2011). 21506742 10.1056/NEJMra1010172 PMC3640822

[b5] Gerdes J. M., Davis E. E. & Katsanis N. The vertebrate primary cilium in development, homeostasis, and disease. Cell 137, 32–45 (2009). 19345185 10.1016/j.cell.2009.03.023 PMC3016012

[b6] Kee H. L. . A size-exclusion permeability barrier and nucleoporins characterize a ciliary pore complex that regulates transport into cilia. Nat. Cell Biol. 14, 431–437 (2012). 22388888 10.1038/ncb2450 PMC3319646

[b7] Lin Y. C. . Chemically inducible diffusion trap at cilia reveals molecular sieve-like barrier. Nat. Chem. Biol. 9, 437–443 (2013). 23666116 10.1038/nchembio.1252 PMC3870470

[b8] Breslow D. K., Koslover E. F., Seydel F., Spakowitz A. J. & Nachury M. V. An in vitro assay for entry into cilia reveals unique properties of the soluble diffusion barrier. J. Cell Biol. 203, 129–147 (2013). 24100294 10.1083/jcb.201212024 PMC3798247

[b9] Rosenbaum J. L. & Witman G. B. Intraflagellar transport. Nat. Rev. Mol. Cell Biol. 3, 813–825 (2002). 12415299 10.1038/nrm952

[b10] Reiter J. F., Blacque O. E. & Leroux M. R. The base of the cilium: roles for transition fibres and the transition zone in ciliary formation, maintenance and compartmentalization. EMBO Rep. 13, 608–618 (2012). 22653444 10.1038/embor.2012.73 PMC3388784

[b11] Schouteden C., Serwas D., Palfy M. & Dammermann A. The ciliary transition zone functions in cell adhesion but is dispensable for axoneme assembly in C. elegans. J. Cell Biol. 210, 35–44 (2015). 26124290 10.1083/jcb.201501013 PMC4493997

[b12] Prevo B., Mangeol P., Oswald F., Scholey J. M. & Peterman E. J. Functional differentiation of cooperating kinesin-2 motors orchestrates cargo import and transport in *C. elegans* cilia. Nat. Cell Biol. 17, 1536–1545 (2015). 26523365 10.1038/ncb3263

[b13] Anderson R. G. The three-dimensional structure of the basal body from the rhesus monkey oviduct. J. Cell Biol. 54, 246–265 (1972). 5064817 10.1083/jcb.54.2.246 PMC2108883

[b14] Graser S. . Cep164, a novel centriole appendage protein required for primary cilium formation. J. Cell Biol. 179, 321–330 (2007). 17954613 10.1083/jcb.200707181 PMC2064767

[b15] Jakobsen L. . Novel asymmetrically localizing components of human centrosomes identified by complementary proteomics methods. EMBO J. 30, 1520–1535 (2011). 21399614 10.1038/emboj.2011.63 PMC3102290

[b16] Tanos B. E. . Centriole distal appendages promote membrane docking, leading to cilia initiation. Genes Dev. 27, 163–168 (2013). 23348840 10.1101/gad.207043.112 PMC3566309

[b17] Wei Q. . Transition fibre protein FBF1 is required for the ciliary entry of assembled intraflagellar transport complexes. Nat. Commun. 4, 2750 (2013). 24231678 10.1038/ncomms3750 PMC3856926

[b18] Sillibourne J. E. . Assessing the localization of centrosomal proteins by PALM/STORM nanoscopy. Cytoskeleton (Hoboken) 68, 619–627 (2011). 21976302 10.1002/cm.20536

[b19] Joo K. . CCDC41 is required for ciliary vesicle docking to the mother centriole. Proc. Natl Acad. Sci. USA 110, 5987–5992 (2013). 23530209 10.1073/pnas.1220927110 PMC3625310

[b20] Gupta G. D. . A dynamic protein interaction landscape of the human centrosome-cilium interface. Cell 163, 1484–1499 (2015). 26638075 10.1016/j.cell.2015.10.065 PMC5089374

[b21] Wei Q., Ling K. & Hu J. The essential roles of transition fibers in the context of cilia. Curr. Opin. Cell Biol. 35, 98–105 (2015). 25988548 10.1016/j.ceb.2015.04.015 PMC4529799

[b22] Schmidt K. N. . Cep164 mediates vesicular docking to the mother centriole during early steps of ciliogenesis. J. Cell Biol. 199, 1083–1101 (2012). 23253480 10.1083/jcb.201202126 PMC3529528

[b23] Lu Q. . Early steps in primary cilium assembly require EHD1/EHD3-dependent ciliary vesicle formation. Nat. Cell Biol. 17, 228–240 (2015). 25686250 10.1038/ncb3109 PMC4344897

[b24] Ye X., Zeng H., Ning G., Reiter J. F. & Liu A. C2cd3 is critical for centriolar distal appendage assembly and ciliary vesicle docking in mammals. Proc Natl Acad Sci USA 111, 2164–2169 (2014). 24469809 10.1073/pnas.1318737111 PMC3926046

[b25] Chaki M. . Exome capture reveals ZNF423 and CEP164 mutations, linking renal ciliopathies to DNA damage response signalling. Cell 150, 533–548 (2012). 22863007 10.1016/j.cell.2012.06.028 PMC3433835

[b26] Failler M. . Mutations of CEP83 cause infantile nephronophthisis and intellectual disability. Am. J. Hum. Genet. 94, 905–914 (2014). 24882706 10.1016/j.ajhg.2014.05.002 PMC4121475

[b27] Adly N., Alhashem A., Ammari A. & Alkuraya F. S. Ciliary genes TBC1D32/C6orf170 and SCLT1 are mutated in patients with OFD type IX. Hum. Mutat. 35, 36–40 (2014). 24285566 10.1002/humu.22477

[b28] Thauvin-Robinet C. . The oral-facial-digital syndrome gene C2CD3 encodes a positive regulator of centriole elongation. Nat. Genet. 46, 905–911 (2014). 24997988 10.1038/ng.3031 PMC4120243

[b29] Ferrante M. I. . Characterization of the OFD1/Ofd1 genes on the human and mouse sex chromosomes and exclusion of Ofd1 for the Xpl mouse mutant. Genomics 81, 560–569 (2003). 12782125 10.1016/s0888-7543(03)00091-0

[b30] Goetz S. C., Liem K. F. Jr & Anderson K. V. The spinocerebellar ataxia-associated gene Tau tubulin kinase 2 controls the initiation of ciliogenesis. Cell 151, 847–858 (2012). 23141541 10.1016/j.cell.2012.10.010 PMC3496184

[b31] Blacque O. E. . Functional genomics of the cilium, a sensory organelle. Curr. Biol. 15, 935–941 (2005). 15916950 10.1016/j.cub.2005.04.059

[b32] Efimenko E. . Analysis of xbx genes in *C. elegans*. Development 132, 1923–1934 (2005). 15790967 10.1242/dev.01775

[b33] Dammermann A. . The hydrolethalus syndrome protein HYLS-1 links core centriole structure to cilia formation. Genes Dev. 23, 2046–2059 (2009). 19656802 10.1101/gad.1810409 PMC2751977

[b34] Evron T . Growth Arrest Specific 8 (Gas8) and G protein-coupled receptor kinase 2 (GRK2) cooperate in the control of Smoothened signalling. J. Biol. Chem. 286, 27676–27686 (2011). 21659505 10.1074/jbc.M111.234666 PMC3149358

[b35] Jeanson L. . Mutations in GAS8, a gene encoding a nexin-dynein regulatory complex subunit, cause primary ciliary dyskinesia with axonemal disorganization. Hum. Mutat. 37, 776–785 (2016). 27120127 10.1002/humu.23005

[b36] Olbrich H. . Loss-of-function GAS8 mutations cause primary ciliary dyskinesia and disrupt the nexin-dynein regulatory complex. Am. J. Hum. Genet. 97, 546–554 (2015). 26387594 10.1016/j.ajhg.2015.08.012 PMC4596893

[b37] Acs P . A novel form of ciliopathy underlies hyperphagia and obesity in Ankrd26 knockout mice. Brain Struct. Funct. 220, 1511–1528 (2015). 24633808 10.1007/s00429-014-0741-9 PMC4601608

[b38] Mee L. . Hydrolethalus syndrome is caused by a missense mutation in a novel gene HYLS1. Hum. Mol. Genet. 14, 1475–1488 (2005). 15843405 10.1093/hmg/ddi157

[b39] Hedgecock E. M., Culotti J. G., Thomson J. N. & Perkins L. A. Axonal guidance mutants of *Caenorhabditis elegans* identified by filling sensory neurons with fluorescein dyes. Dev. Biol. 111, 158–170 (1985). 3928418 10.1016/0012-1606(85)90443-9

[b40] Perkins L. A., Hedgecock E. M., Thomson J. N. & Culotti J. G. Mutant sensory cilia in the nematode *Caenorhabditis elegans*. Dev. Biol. 117, 456–487 (1986). 2428682 10.1016/0012-1606(86)90314-3

[b41] Doroquez D. B., Berciu C., Anderson J. R., Sengupta P. & Nicastro D. A high-resolution morphological and ultrastructural map of anterior sensory cilia and glia in *Caenorhabditis elegans*. Elife 3, e01948 (2014). 24668170 10.7554/eLife.01948 PMC3965213

[b42] Wei Q. . The BBSome controls IFT assembly and turnaround in cilia. Nat. Cell Biol. 14, 950–957 (2012). 22922713 10.1038/ncb2560 PMC3434251

[b43] Williams C. L. . MKS and NPHP modules cooperate to establish basal body/transition zone membrane associations and ciliary gate function during ciliogenesis. J. Cell Biol. 192, 1023–1041 (2011). 21422230 10.1083/jcb.201012116 PMC3063147

[b44] Huang L. . TMEM237 is mutated in individuals with a Joubert syndrome related disorder and expands the role of the TMEM family at the ciliary transition zone. Am. J. Hum. Genet. 89, 713–730 (2011). 22152675 10.1016/j.ajhg.2011.11.005 PMC3234373

[b45] Roberson E. C. . TMEM231, mutated in orofaciodigital and Meckel syndromes, organizes the ciliary transition zone. J. Cell Biol. 209, 129–142 (2015). 25869670 10.1083/jcb.201411087 PMC4395494

[b46] Williams C. L., Winkelbauer M. E., Schafer J. C., Michaud E. J. & Yoder B. K. Functional redundancy of the B9 proteins and nephrocystins in *Caenorhabditis elegans* ciliogenesis. Mol. Biol. Cell 19, 2154–2168 (2008). 18337471 10.1091/mbc.E07-10-1070 PMC2366840

[b47] Williams C. L., Masyukova S. V. & Yoder B. K. Normal ciliogenesis requires synergy between the cystic kidney disease genes MKS-3 and NPHP-4. J. Am. Soc. Nephrol. 21, 782–793 (2010). 20150540 10.1681/ASN.2009060597 PMC2865747

[b48] Singla V., Romaguera-Ros M., Garcia-Verdugo J. M. & Reiter J. F. Ofd1, a human disease gene, regulates the length and distal structure of centrioles. Dev. Cell 18, 410–424 (2010). 20230748 10.1016/j.devcel.2009.12.022 PMC2841064

[b49] Ishikawa H., Kubo A. & Tsukita S. Odf2-deficient mother centrioles lack distal/subdistal appendages and the ability to generate primary cilia. Nat. Cell Biol. 7, 517–524 (2005). 15852003 10.1038/ncb1251

[b50] Wang C., Low W. C., Liu A. & Wang B. Centrosomal protein DZIP1 regulates Hedgehog signalling by promoting cytoplasmic retention of transcription factor GLI3 and affecting ciliogenesis. J. Biol. Chem. 288, 29518–29529 (2013). 23955340 10.1074/jbc.M113.492066 PMC3795250

[b51] von Tobel L. . SAS-1 is a C2 domain protein critical for centriole integrity in *C. elegans*. PLoS Genet. 10, e1004777 (2014). 25412110 10.1371/journal.pgen.1004777 PMC4238951

[b52] Brenner S. The genetics of *Caenorhabditis elegans*. Genetics 77, 71–94 (1974). 4366476 10.1093/genetics/77.1.71 PMC1213120

[b53] Hall D. H. Electron microscopy and three-dimensional image reconstruction. Methods Cell Biol. 48, 395–436 (1995). 8531736 10.1016/s0091-679x(08)61397-7

