# Supplementary material for: The hydrolethalus syndrome protein HYLS-1 regulates formation of the ciliary gate
Source: Nat Commun. 2016 Aug 18;7:12437. doi: 10.1038/ncomms12437 (PMC4992140; doi:10.1038/ncomms12437)
Supplement: Supplementary Information — Supplementary Figures 1-4, Supplementary Tables 1 & 2 [file ncomms12437-s1.pdf]

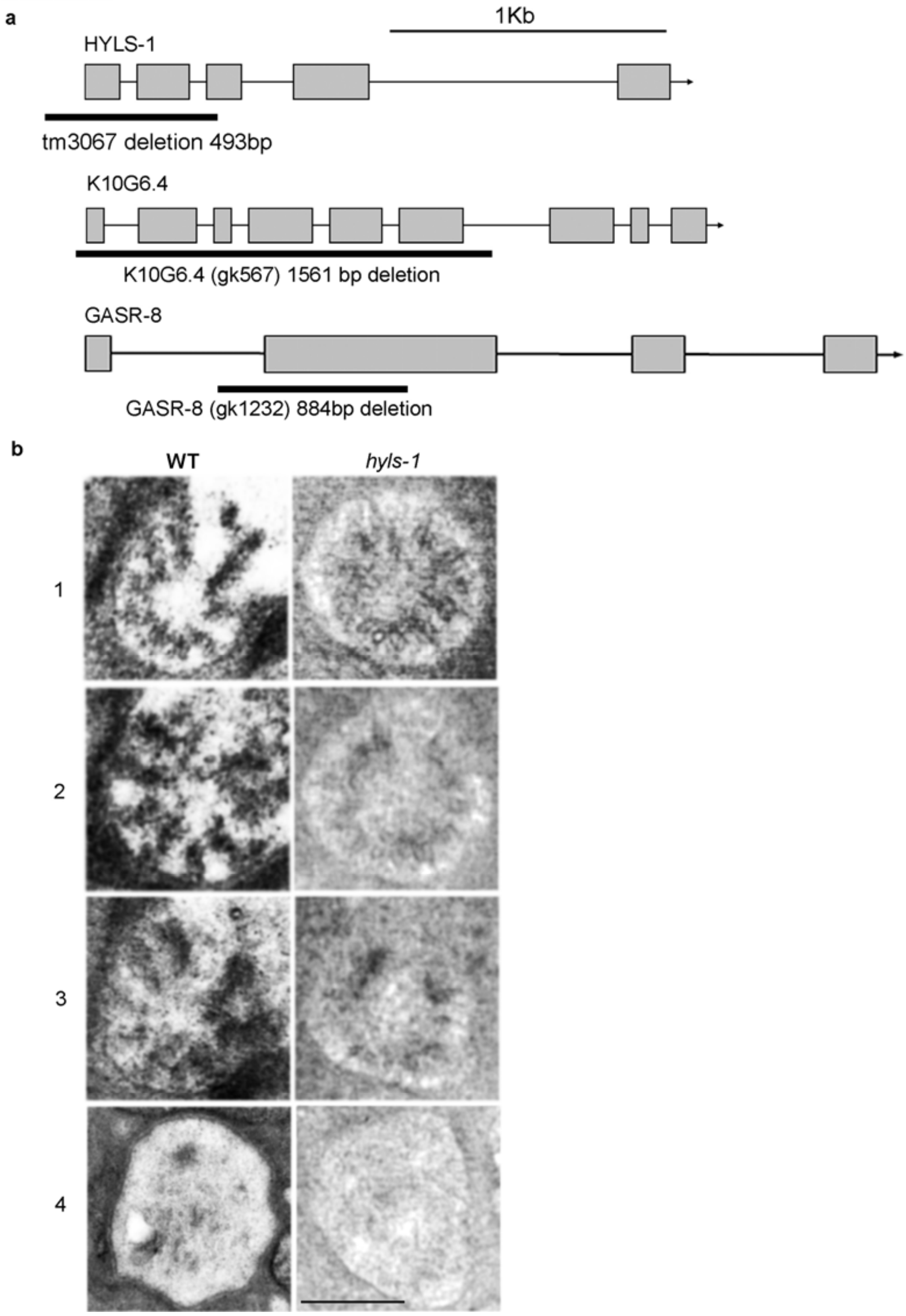

Supplementary Figure 1. Gene schematics of *hyls-1, gasr-8* and *k10g6.4,* and TEM analysis of TFs in WT and *hyls-1* cilia.

(**a**) Gene structure of *hyls-1*, *gasr-8* and *k10g6.4* based on WormBase (http://wormbase.org), indicating available mutants. The *tm3067* allele has a 493bp DNA fragment deletion, encompassing the 1st, 2nd and part of the 3rd exon of the *hyls-1* gene, removing the first 361bp of the 825bp CDS including the ATG. The *gk567* allele has a 1561bp DNA fragment deletion, encompassing exons 1 to 6 of *k10g6.4* and removing the the first 871bp of the 1380bp CDS including the ATG. The *gk1232* allele has an 884bp DNA fragment deletion, which removes exon 2 of *gasr-8* and is predicted to result in an in-frame deletion of 261aa (S28-Q288) of the 410aa wild-type protein. All three alleles are putative *null* due to the large deletion. (**b**) TEM analysis of TFs in WT and *hyls-1* cilia. TEM images of four consecutive serial sections (~80 nm thickness each) of the basal body region from WT or *hyls-1* mutants. Fiber-like structures in similar size can be observed in serial sections of WT cilia. In contrast, only a few areas of electron-dense material but no organized fibers remain in the basal body region of *hyls-1* mutants. Scale bar, 200 nm.

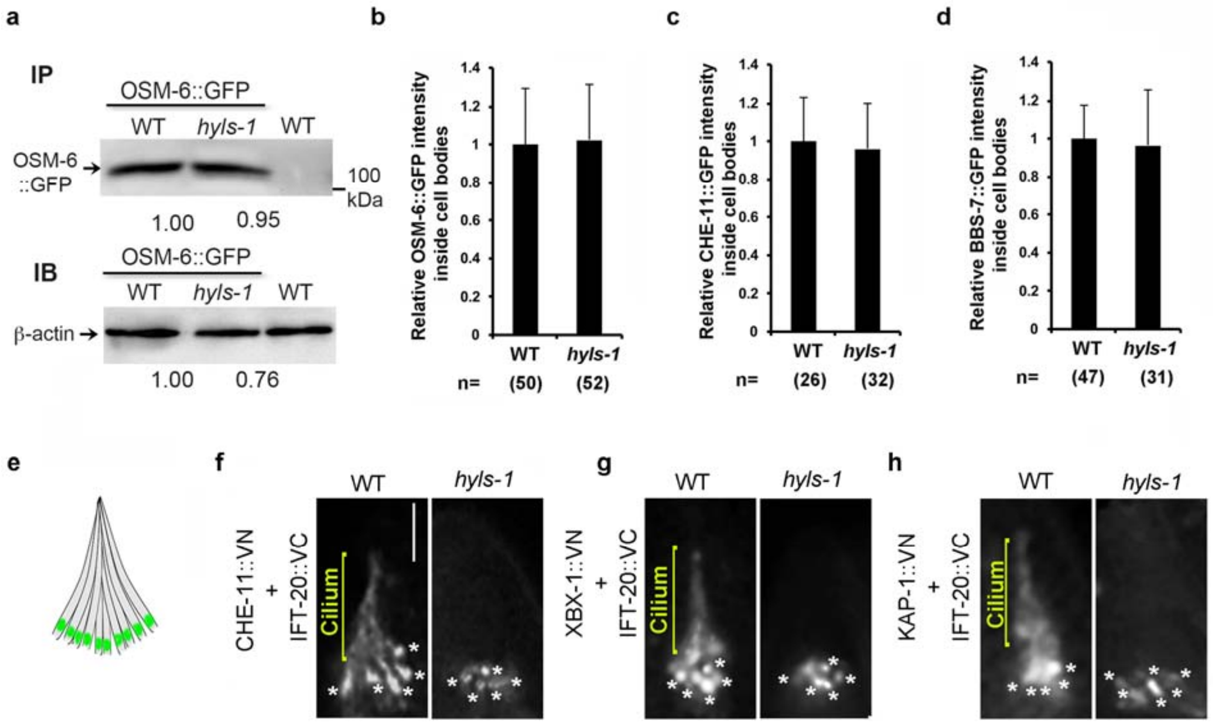

Supplementary Figure 2. Quantitation of the expression level of IFT components, and visualization of IFT-A, IFT-B and motor association in cilia of WT and *hyls-1* mutants. (**a**) Upper panel, OSM-6::GFP was immunoprecipitated from whole worm lysates with anti-GFP monoclonal antibody and then probed with anti-GFP antibody. Lower panel: whole worm lysate was probed with anti-β-Actin (loading control). After normalization against actin levels, OSM-6::GFP shows no difference in expression level in WT and *hyls-1* mutants. (**b-d**) Quantitation of IFT fluorescence intensities in the cell body shows no difference in levels between WT and *hyls-1* mutants. Error bars indicate S.D. *n* represents the number of cilia analyzed. **(e)** Schematic of amphid sensory organ. Each amphid contains ten cilia whose distal segments bundle together. Green dots indicate ciliary base. (**f-h**) All BiFC pairs, CHE-11::VN + IFT-20::VC (**f**), XBX-1::VN + IFT-20::VC (**g**), and KAP-1::VN + IFT-20::VC IFT-A (**h**), show fluorescence complementation in both WT and *hyls-1* amphid cilia, indicating formation of functional IFT complexes. However, fluorescence complementation signal is largely restricted to the cilia base in *hyls-1* mutants. Asterisks indicate the cilia bases. Note that not all ten cilia can be visualized in one focal plane in confocal imaging. VN, N-terminus of Venus. VC, C-terminus of Venus. Scale bar, 5 μm.

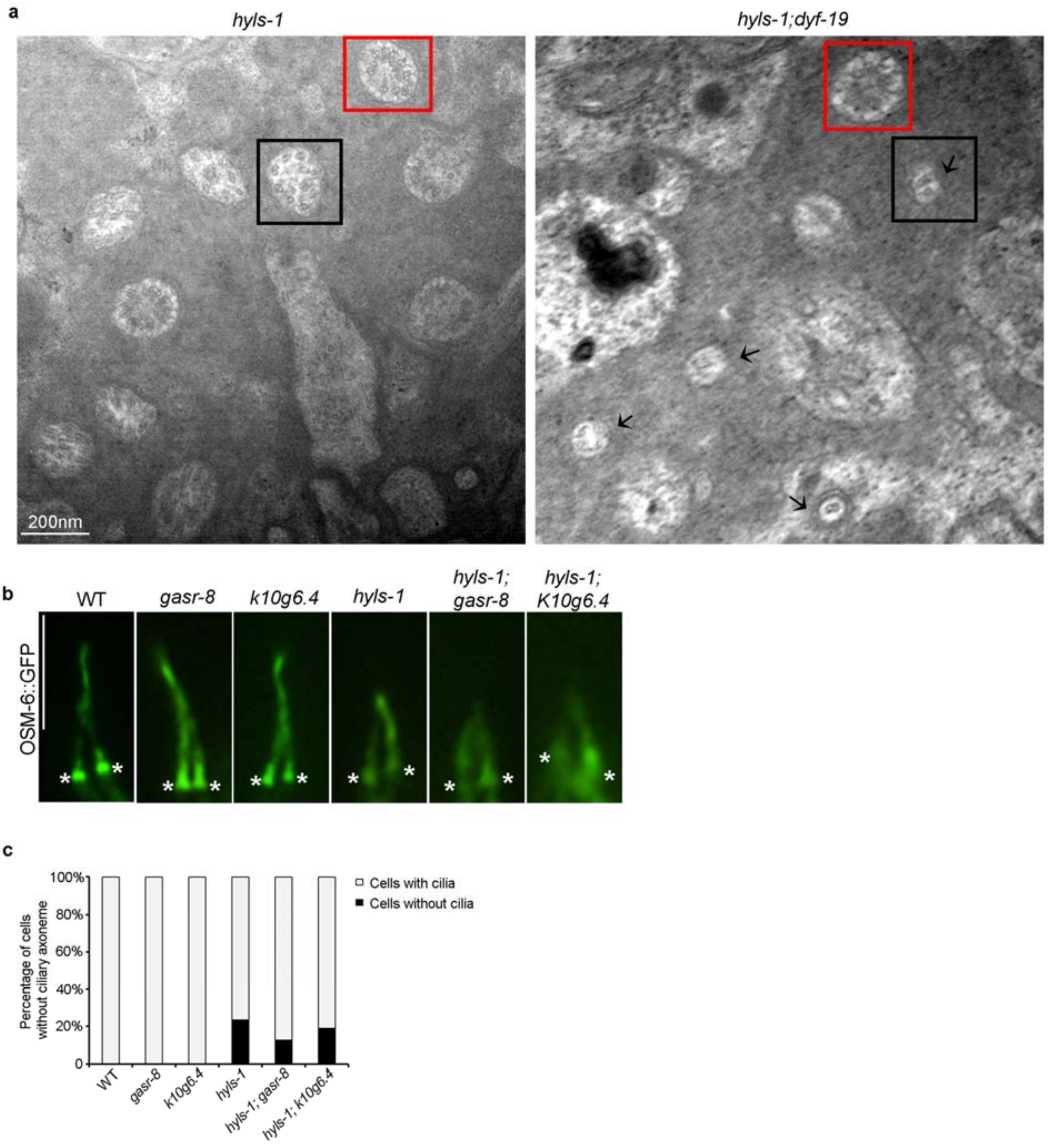

Supplementary Figure 3. Genetic interactions between *hyls-1* and TF mutants.
(a) TEM analysis of amphid sensory cilia of *hyls-1* and *hyls-1;dyf-19* double mutants. Red box indicates a cilium cut at the level of the TZ. Black box indicates axoneme immediately adjacent to the TZ. Compared to *hyls-1* single mutants, most axonemes terminate immediately distal to the TZ in *hyls-1; dyf-19* double mutants (Black arrows). Bar, 200 nm. (**b)** *hyls-1* shows no genetic interactions with *gasr-8* or *k10g6.4* in ciliogenesis. Asterisks indicate cilia base of phasmid cilia expressing the IFT marker OSM-6::GFP. Scale bar, 5 µm. **(c)** Quantification of ciliation in phasmid neurons based on TBB-4::mCherry in different genetic backgrounds, >100 phasmid neurons analyzed for each genetic background.

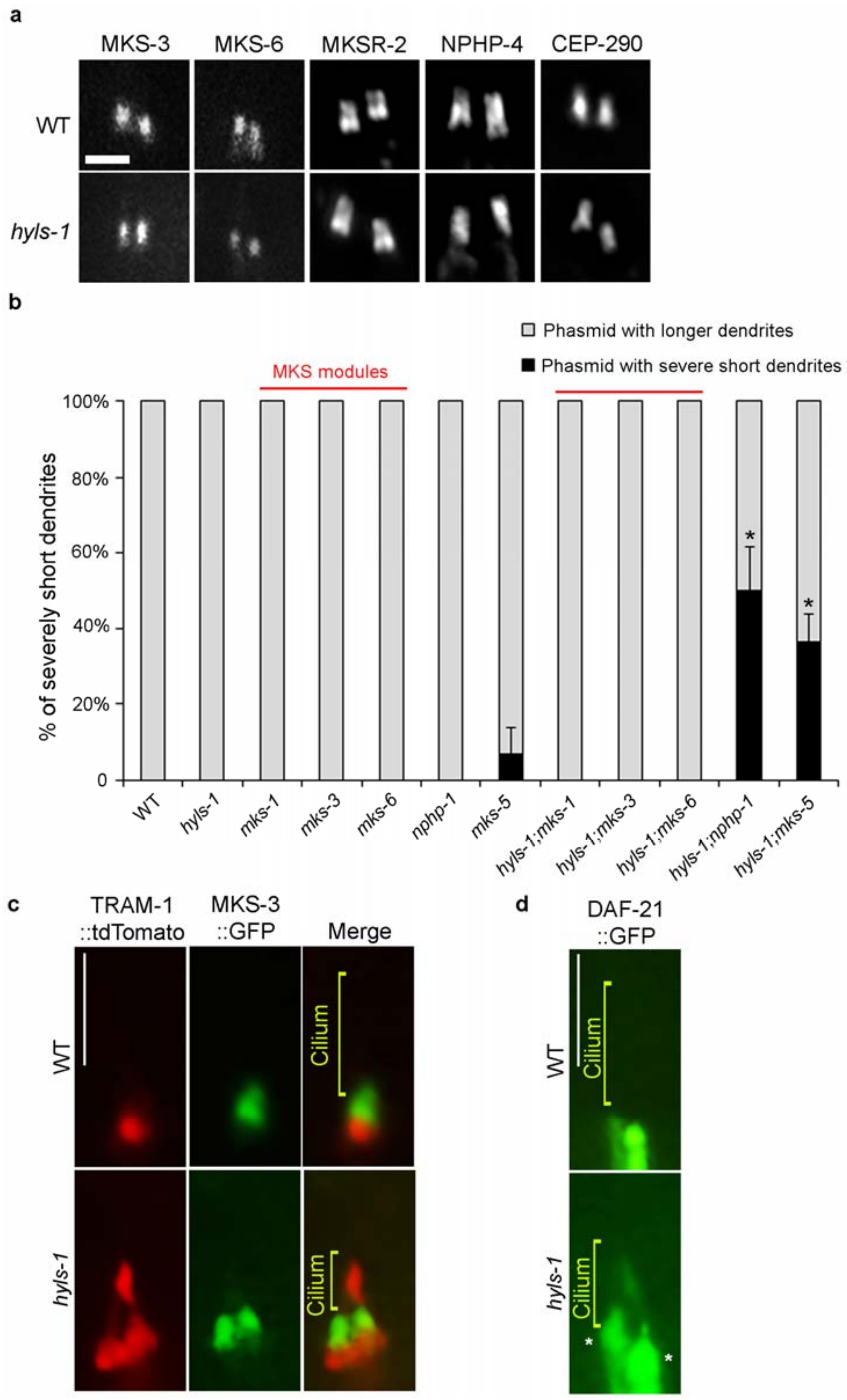

Supplementary Figure 4. Genetic interactions between HYLS-1 and TZ proteins and compromised gating in HYLS-1-deficient cilia.

(**a**) Localization of TZ components in *hyls-1* mutants. HYLS-1 is not required for proper TZ localization of MKS-3, MKS-6, MKSR-2, NPHP-4, and CCEP-290. Scale bar, 1 µm. (**b**) Quantification of dendrite collapse in double mutants of *hyls-1* and various TZ genes. Severe shortened dendrites were observed in *hyls-1; nphp-1* and *hyls-1; mks-5* double mutants. No genetic interaction was observed between *hyls-1* and MKS module mutants (*mks-1, mks-3 or mks-6*). > 100 phasmid neurons analyzed for each genetic background. Error bars indicate S.D. Student's t-test for significant differences, * P<0.001. (**c**) tdTomato-tagged non-ciliary membrane protein TRAM-1 leaks into phasmid cilia in *hyls-1* mutants. GFP-tagged MKS-3 was used as a marker to label the TZ. Scale bar, 5 µm. (**d**) Non-ciliary cytoplasmic protein DAF-21 leaks into phasmid cilia in *hyls-1* mutants. Scale bar, 5 µm.

Supplementary Table 1. Quantitative Analysis of Transition Fibers (TFs) and Transition Zone (TZ) in serial section TEM study.

		WT	*hyls-1*	*dyf-19*	*hyls-1; dyf-19*
Number of cilia examined		19	16	8	14
TF	Number of TFs with fiber-like structures	19	2 (*)	8	0
	Percentage of TFs with fiber-like structures	100%	12.5% (*)	100%	0
TZ	Number of TZ with Y-links	10	16	8	14
	Percentage of TZ with Y-links	100%	100%	100%	100%

*: the fiber-like structure observed in *hyls-1* mutants is not intact.

Supplementary Table 2. ***C. elegans*** **strains used in this study**

Strain name	Genotype
OD192	*hyls-1(tm3067)V*
ZP541	*dyf-19(jhu455)V*
VC2343	*gasr-8 (gk1232)V*
VC1268	*k10g6.4 (gk567)II*
FX2705	*mks-1 (tm2705)III*
FX2547	*mks-3 (tm2547)II*
FX3100	*mks-5 (tm3100)II*
RB743	*nphp-1(ok500)II*
VC1466	*mks-6(gk674)I*
ZP2174	*mks-1 (tm2705)III*; *hyls-1(tm3067)V*; IsOSM6
ZP2175	*mks-6(gk674)I*; *hyls-1(tm3067)V*; IsOSM6
ZP2176	*nphp-1(ok500)II*; *hyls-1(tm3067)V*; IsOSM6
ZP2177	*gasr-8 (gk1232)V*; *hyls-1(tm3067)V*; IsOSM6
ZP2203	*dyf-19(jhu455)V*; *hyls-1(tm3067)V*; IsOSM-6
ZP2204	*k10g6.4 (gk567)II*; *hyls-1(tm3067)V*; IsOSM-6
ZP2205	*mks-5 (tm3100)II*; *hyls-1(tm3067)V*; IsOSM-6
ZP2179	*mks-3 (tm2547)II* ; *hyls-1(tm3067)V*; IsOSM6
ZP1389	*jhuEx [HYLS1:GFP+DYF-9:mCherry+pRF4]*
ZP1993	*jhuEx [FBF1::mCherry+GASR-8::GFP+pRF4]*
ZP1997	*jhuEx [NPHP1::GFP+GASR-8::mCherry+pRF4]*
ZP2001	*jhuEx [NPHP1::GFP+FBF1::mCherry+pRF4]*
ZP1880	*jhuEx [K10G6.4::GFP+DYF-9::mCherry+pRF4]*
SP2101	*mnIs17 [OSM6::GFP]*
ZP1986	*hyls-1(tm3067)V*; IsOSM-6
ZP2035	*hyls-1(tm3067)V*; *Ex [CHE-11::GFP+pRF4]*
PT50	*che-11(e1810)V; Ex [CHE-11::GFP+pRF4]*
ZP1205	*jhuEx [BBS-7::GFP +pRF4]*
ZP1397	*jhuEx [BBS-7::GFP+MKS-5::mCherry+pRF4]*

ZP2156	*hyls-1(tm3067)V*; *jhuEx [BBS-7::GFP +pRF4]*
ZP2180	*hyls-1(tm3067)V*; *jhuEx [BBS-7::GFP+MKS-5::mCherry+pRF4]*
YH490	*yhEx [MKS-1::YFP+CHE-13::CFP+pRF4]*
YH751	*yhEx [MKS-6::GFP+XBX-1::tdTomao+pRF4]*
YH652	*yhEx [GFP::MKS-3+XBX-1::tdTomato+pRF4]*
ZP2024	*hyls-1(tm3067)V*; *jhuEx [TBB-4::mCherry+DYF-19::GFP+pRF4]*
ZP2025	*hyls-1(tm3067)V*; *yhEx [GFP::MKS-3+XBX-1::tdTomato+pRF4]*
ZP2026	*hyls-1(tm3067)V*; *yhEx [MKS-1::YFP+CHE-13::CFP+pRF4]*
ZP2027	*jhuEx [TBB-4::mCherry+DYF-19::GFP+pRF4]*
ZP2032	*hyls-1(tm3067)V*; *jhuEx [NPHP1::GFP+FBF1::mCherry+pRF4]*
ZP2033	*hyls-1(tm3067)V*; *jhuEx [NPHP1::GFP+Gasr-8::mCherry+pRF4]*
ZP1855	*hyls-1(tm3067)V*; *jhuEx [RPI-2::GFP+MKS-5::mCherry+pRF4]*
ZP1170	*jhuEx [CHE-11::Venus1-173+IFT-20::Venus155-238+pRF4]*
ZP1346	*hyls-1(tm3067)V*; *jhuEx [CHE-11::Venus1-173+IFT-20::Venus155-238+pRF4]*
ZP2291	*jhuEx [XBX-1::Venus1-173+IFT-20::Venus155-238+pRF4]*
ZP2299	*hyls-1(tm3067)V*; *jhuEx [XBX-1::Venus1-173+IFT-20::Venus155-238+pRF4]*
ZP2290	*jhuEx [KAP-1::Venus1-173+IFT-20::Venus155-238+pRF4]*
ZP2292	*hyls-1(tm3067)V*; *jhuEx [KAP-1::Venus1-173+IFT-20::Venus155-238+pRF4]*
ZP2293	*jhuEx [OSM-3::Venus1-173+IFT-20::Venus155-238+pRF4]*
ZP2294	*hyls-1(tm3067)V*; *jhuEx [OSM-3::Venus1-173+IFT-20::Venus155-238+pRF4]*
ZP2285	*jhuEx[F29G9.2::GFP+MKS-5::mCherry+pRF4]*
ZP2287	*hyls-1(tm3067)V*; *jhuEx[F29G9.2::GFP+MKS-5::mCherry+pRF4]*
YH670	*yhEx[TRAM-1::tdTomato+MKS-3::GFP+pRF4]*
ZP2297	*hyls-1(tm3067)V; yhEx[TRAM-1::tdTomato+MKS-3::GFP+pRF4]*
ZP2056	*jhuEx[Posm-9::OSM-9::GFP+pRF4]*
ZP2296	*hyls-1(tm3067)V*; *jhuEx[Posm-9::OSM-9::GFP+pRF4]*
ZP2289	*jhuEx[Parl-13::DAF-21::GFP+pRF4]*
ZP2298	*hyls-1(tm3067)V*; *jhuEx[Parl-13::DAF-21::GFP+pRF4]*
DAM278	*vieSi12[GFP::CCEP-290; cb unc-119(+)]*
DAM323	*vieSi22[GFP::MKSR-2; cb unc-119(+)]*
DAM324	*vieSi23[GFP::NPHP-4; cb unc-119(+)]*
